# Bent not broken

**DOI:** 10.1002/jhm.70200

**Published:** 2025-10-07

**Authors:** Gabrielle Rogie

**Affiliations:** ^1^ Texas A&M School of Engineering Medicine (EnMed) Houston Texas USA

I was 12 when a routine school screening upended my sense of normal. The nurse had me bend forward, paused, and asked me to repeat the motion. I knew something was wrong the moment she took longer than for my peers. A week later, I sat in an orthopedist's office, staring at an X‐ray of a spine bent like a question mark. I heard words like “Risser 4” and “27‐degree Cobb angle” but didn't know what any of these words meant. I only knew that my curve was not severe enough for a brace.

So, we waited.

Those years of “watchful waiting” were anything but passive. Each follow‐up felt like spinning a roulette wheel, a lesson I now carry into my clinical practice. I know that even when the body is at a standstill, the mind is not. A steady current of anxiety ran beneath the surface of my daily life, a silent calculation of hopes and dreams. Furthermore, without a brace, I appeared “normal,” but I obsessed over posture, clothing, and symmetry. I now understand that for patients, “watchful waiting” is a period filled with silent calculations, hopes, and dreams, not a passive one.

Scoliosis shapes adolescence in visible and invisible ways. More than the curve itself, it's the feeling of misalignment, physical and emotional, that is heavy. Teenagers already struggle to fit in with their peers‐ adding one more thing to make them feel different is a significant burden. These are the things that need to be considered in shared decision making when it comes to treatment. To recognize this as a provider is to honor both the body and the person.

By the time I reached medical school, my curve had worsened to the point where surgery was no longer hypothetical but necessary. I had spent over a decade in a body that felt increasingly asymmetrical, increasingly unpredictable. And now, in the midst of dissecting cadavers and studying pathophysiology, I became a patient again. People often ask if I still have scoliosis. I tell them that I will always have scoliosis. Even after surgery, my anxiety still lingers quietly in the corner of every room, asking, “What if it's getting worse?”.

Support for the person can be found outside the clinical setting. For me, finding Curvy Girls Scoliosis, an international support group system for girls living with the condition, changed my entire perspective. It was the first place I met others who understood—who didn't ask what scoliosis was or look surprised when I talked about X‐rays, physical limitations, or the emotional toll. At meetings, I heard stories from girls who felt they had nowhere to turn. They would cry and confess to me how alone they felt, a feeling I knew all too well. I met one girl who was so terribly bullied for wearing her brace. At the annual Curvy Girls convention, she stood among a hundred other girls just like her and, for the first time, felt she wasn't alone. Empowered, she left the convention wearing her brace on the outside of her clothes. These girls have been my biggest inspiration in life, and my involvement highlighted the importance of peer support to patients.

I carried these lessons with me into my medical school training. During my pediatric orthopedic rotation, I met a young girl newly diagnosed with scoliosis—already at a surgical threshold. She sat curled in the exam chair, eyes downcast, while her mother asked precise questions about degrees, curves, and prognosis. I saw their worry mirrored in one another and felt a familiar pang. It was the feeling of physical and emotional misalignment that had weighed on me for years. As I left the clinic, I saw the mother and daughter in the lobby, both with red eyes. Though nervous, I knew I had to speak. I told the girl that I had scoliosis, too, and watched her look up at me—truly look at me—for the first time. “And you're going to be a doctor?” she asked, her face lighting up. In that moment, I saw the power of representation. I saw how a diagnosis can make a teenager question everything they thought was possible. It was a powerful reminder that our role is not only to correct anatomy but to protect the personhood that lives within it. This means inviting patients to share their fears and expectations, acknowledging uncertainty openly, and connecting them with support networks early.

In the clinic or the operating room, I often saw a younger version of myself: scared, uncertain, and waiting to hear whether my body would cooperate or betray me. I grieved for what I had lost, but also for what scoliosis quietly steals from so many young girls—confidence, ease, and the simple luxury of not thinking about your back every waking moment. It is a thief of childhood, in invisible ways. As that grief softened, a commitment grew in its place. A commitment to ask not only “Where does it hurt?” but also, “What has this meant to you?” Because the answers to both questions are essential to care.

As physicians, we often speak of objectivity and evidence. These matter, but so does the unmeasurable‐ the meaning patients assign to their diagnosis, the ways it reshapes identity, confidence, and daily life. To treat the spine without attending to the self is to miss part of the pathology. I do not romanticize my scoliosis. It left both physical and emotional scars. But it also gave me fluency—the ability to translate the lived experience of illness into a language patients and physicians can share. That fluency now guides how I listen, how I advocate, and how I show up for my patients. Our role is not only to correct anatomy but to protect the personhood that lives within it (Figure 1).

**Figure 1:**
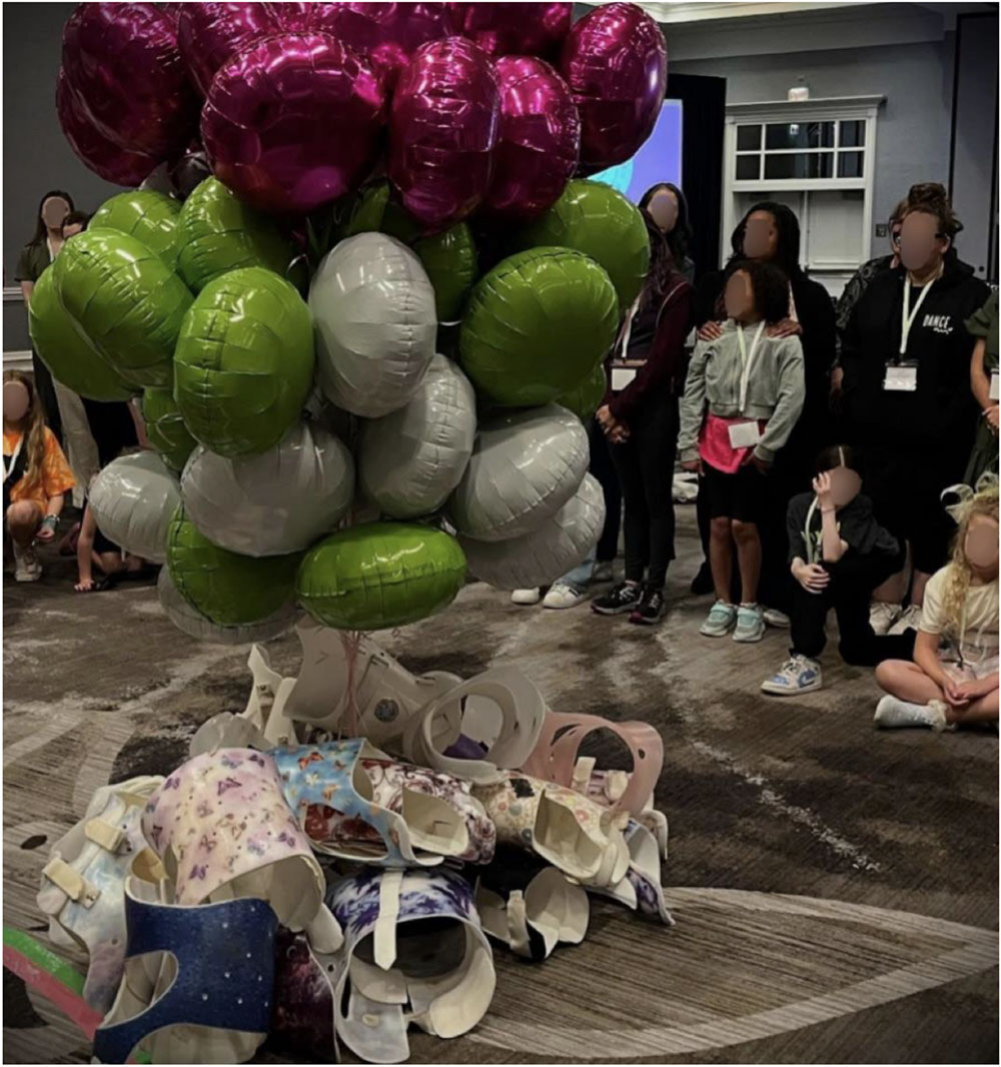
The blessing of donated braces—Curvy Girls Scoliosis Convention 2025.

## CONFLICT OF INTEREST STATEMENT

The author declares no conflict of interest.

